# Rodent-targeted approaches to reduce acarological risk of human exposure to pathogen-infected *Ixodes* ticks

**DOI:** 10.1016/j.ttbdis.2023.102119

**Published:** 2023-01-14

**Authors:** Lars Eisen

**Affiliations:** Division of Vector-Borne Diseases, National Center for Emerging and Zoonotic Infectious Diseases, Centers for Disease Control and Prevention, 3156 Rampart Road, Fort Collins, CO 80521, United States

**Keywords:** *Ixodes*, Pathogen, Rodent, Tick, Control

## Abstract

In the United States, rodents serve as important hosts of medically important *Ixodes* ticks, including *Ixodes scapularis* and *Ixodes pacificus*, as well as reservoirs for human pathogens, including *Anaplasma phagocytophilum, Borrelia burgdorferi* sensu stricto (s.s.), and *Babesia microti*. Over the last four decades, different methods to disrupt enzootic transmission of these pathogens between tick vectors and rodent reservoirs have been developed and evaluated. Early work focused on self-application of topical acaricide by rodents to kill infesting ticks; this resulted in two different types of commercial products based on (i) delivery of permethrin to rodents via impregnated cotton offered as nesting material or (ii) application of fipronil to rodents via an impregnated wick as they navigate through a bait box to reach a food source. More recent work has focused on approaches where acaricides, antibiotics, or a vaccine against *Bo. burgdorferi* s.s. are delivered orally via rodent food baits. Of these, the oral vaccine and oral acaricide are nearest to commercialization. Other approaches in early stages of development include anti-tick vaccines for rodents and use of heritable genome editing to engineer white-footed mice (*Peromyscus leucopus*) that are refractory to *Bo. burgdorferi* s.s. In this review, I first outline general benefits and drawbacks of rodent-targeted tick and pathogen control methods, and then describe the empirical evidence for different approaches to impact enzootic pathogen transmission and acarological risk of human exposure to pathogen-infected *Ixodes* ticks. Rodent-targeted methods remain promising components of integrated tick management approaches but there are concerns about the robustness of the impact of existing rodent-targeted products across habitats and variable tick host communities, and in some cases also for the implementation cost in relation to what homeowners in Lyme disease endemic areas say they are willing to pay for tick control.

## 1. Background

Rodents were implicated as enzootic reservoirs of Lyme disease spirochetes, *Borrelia burgdorferi* sensu lato (s.l.), within a few years of the realization that the blacklegged tick, *Ixodes scapularis*, is the primary vector of these microorganisms to humans in the eastern United States (Burgdorfer et al., 1982; Levine et al., 1985; Donahue et al., 1987). The white-footed mouse, *Peromyscus leucopus*, was identified as a primary reservoir for *Bo. burgdorferi* s.l. (Mather et al., 1989), in addition to its previously known role as a reservoir for another human pathogen transmitted by *Ix. scapularis*: the protozoan babesiosis agent, *Babesia microti* (Healy et al., 1976; Spielman, 1976; Spielman et al., 1981). Later studies have reinforced the importance of *Peromyscus* mice as reservoirs of *Ba. microti* and *Bo. burgdorferi* s.l., including for two Lyme disease agents within this species complex: *Borrelia burgdorferi* sensu stricto (s. s.) and *Borrelia mayonii* (reviewed by Tsao et al. 2021). Rodents were also shown to be involved in enzootic maintenance of additional *Ix. scapularis*-borne human pathogens, including bacterial agents causing anaplasmosis (*Anaplasma phagocytophilum*), relapsing fever (*Borrelia miyamotoi*), and ehrlichiosis (*Ehrlichia muris eauclairensis*) (Telford et al., 1996; Walls et al., 1997; Levin et al., 2002; Barbour et al., 2009; Castillo et al., 2015; Johnson et al., 2017; Larson et al., 2021). In this paper, I use *Bo. burgdorferi* s.s. for instances when an isolate known to represent this species was used in a laboratory study or when the pathogen detection methodology used for field samples was stated to separate *Bo. burgdorferi* s.s. from other species within the *Bo. burgdorferi* s.l. complex present in the United States. For studies where it is not clear if the pathogen detection methodology could distinguish *Bo. burgdorferi* s.s., including the seminal studies on rodent reservoirs from the 1980s mentioned above, I conservatively use *Bo. burgdorferi* s.l.

The developing knowledge of how *Ba. microti* and *Bo. burgdorferi* s.l. are maintained in nature led to the realization in the 1980s that targeting the rodent pathogen reservoir potentially could reduce the intensity of enzootic transmission and thus decrease the abundance of questing infected *Ix. scapularis* in the environment posing a threat to humans (Spielman, 1988; Anderson, 1989; Stafford, 1989). The concept of self-application by rodents with topical acaricide to kill infesting ticks had already been explored for the American dog tick, *Dermacentor variabilis*, using a “baited pesticide treatment station” (Sonenshine and Haines, 1985), inspired by earlier work on control of fleas on rodents (Kartman, 1958, 1960; Barnes and Kartman, 1960; Barnes et al., 1974). Initial work on self-application of topical acaricide by rodents specifically targeting the two primary vectors of Lyme disease spirochetes to humans in the United States (*Ix. scapularis* and the closely related western blacklegged tick, *Ixodes pacificus*) focused on development and field evaluation of a novel methodology where cotton treated with a synthetic pyrethroid (permethrin) was presented as nesting material for *Peromyscus* mice and other rodents with the aim of killing infesting ticks (reviewed in Section 3.1). Subsequent work on rodent-targeted approaches to kill infesting *Ixodes* ticks and/or disrupt enzootic pathogen transmission has explored a range of different methodologies: rodent self-application of topical acaricide via baited treatment stations (Section 3.2); oral delivery of acaricide to rodents (Section 3.3); oral delivery of antibiotics to cure infection in rodents (Section 3.4); oral delivery of anti-tick vaccine to disrupt tick feeding and prevent pathogen acquisition from and transmission to rodents (Section 3.5); vaccination of rodents against *Bo. burgdorferi* s.s. (Section 3.6), and use of heritable genome editing to engineer white-footed mice that are refractory to *Bo. burgdorferi* s.s. and pass this trait to their offspring (Section 3.7). Current availability of commercial products and expected general outcomes for use of different rodent-targeted approaches, with respect to impacts on rodent reservoirs and *Ixodes* vectors, are outlined in Table 1.

The present paper builds upon a previous exhaustive review by Eisen and Dolan (2016) of environmentally based methods to suppress *Ix. scapularis* and its associated pathogens, including rodent-targeted approaches. It also incorporates additional publications resulting from accelerated research since 2016 on rodent-targeted approaches aiming to suppress pathogen-infected *Ix. scapularis* in the environment, reduce human bites by this tick, and ultimately prevent cases of human illness caused by *Ix. scapularis*-associated pathogens (Dolan et al., 2017; Schulze et al., 2017; Keesing and Ostfeld, 2018; Williams et al., 2018a, 2018b, 2020; Buchtal et al., 2019; Jordan and Schulze, 2019, 2020; Machtinger and Li, 2019; Brown et al., 2020; Carrera-Pineyro et al., 2020; Pelletier et al., 2020, 2022; Poché et al., 2020, 2021; Stafford et al., 2020; Hinckley et al., 2021; Mandli et al., 2021; Phillip et al., 2021; van Oosterwijk and Wikel, 2021; Keesing et al., 2022; Linske et al., 2022). These recently published studies, which include large scale intervention trials that involved rodent-targeted acaricide and assessed human-based outcomes, sets the stage for a fresh assessment of the future of rodent-targeted tick and pathogen control methods.

## 2. General benefits and drawbacks of rodent-targeted approaches to suppress questing pathogen-infected *Ix. scapularis*

Benefits and drawbacks of rodent-targeted approaches were addressed previously in relation to other methods to suppress *Ix. scapularis*, such as vegetation management, broadcast application of acaricides or biological control agents to kill questing ticks, and deer-targeted approaches (Eisen and Dolan, 2016; Eisen and Stafford, 2021). Below, I provide a more in-depth discussion of the benefits and drawbacks specific to rodent-targeted approaches.

### 2.1. General benefits

One general benefit of using a rodent-targeted approach is the spatial extent impacted by the intervention. As the home range of *Peromyscu*s mice averages 0.1 to 1 ha in different environments (Wolff, 1985; Nupp and Swihart, 1996; Gaitan and Millien, 2016), control approaches targeting these mice are well suited for use on residential properties. With strategic placement of treatment delivery devices, it should be possible to impact all individual mice with home ranges falling completely or in part within the boundary of a single property, and thus to achieve reduction in the abundance of questing *Ix. scapularis* across the entire residential property. The importance of an intervention impacting all portions of a residential property where human tick encounters may occur was underscored by a study in the northeastern United States where broadcast application of synthetic acaricide only along the lawn-woods ecotone of residential properties resulted in roughly 60% reduction in the abundance of questing *Ix. scapularis* nymphs specifically within the spray area, but where no reduction was seen for ticks crawling on or biting humans residing on the treated properties (Hinckley et al., 2016). Most human encounters with ticks in the northeastern United States are considered to occur on residential properties (Stafford et al., 2017; Mead et al., 2018; Jordan and Egizi, 2019). The lack of impact on human tick encounters in the study by Hinckley et al. (2016) despite a 60% reduction in questing *Ix. scapularis* nymphs along the residential lawn-woods ecotone was most likely related to only a limited portion of the properties having been treated and tick encounters occurring commonly on the non-treated portions of the properties, which included wooded habitat favorable for *Ix. scapularis*. Alternatively, as suggested by Fischhoff et al. (2019), tick exposures also commonly could have occurred away from residential properties in other frequently used parts of the neighborhood.

A recent study aimed to use self-application of topical acaricide by rodents at a larger neighborhood-wide scale, by attempting to recruit all properties in the included neighborhoods to participate (Keesing and Ostfeld, 2018; Keesing et al., 2022). Such scaling up should increase the probability of the treatment impacting all rodents frequenting a given individual treated property, as it would border on other treated properties rather than non-treated ones. However, even with the intervention provided at no cost to the homeowners as part of a research study, only roughly one third of invited homeowners elected to participate (Keesing et al., 2022). Although theoretically possible to scale up to neighborhoods, approaches based on delivery of acaricides, antibiotics, or vaccines to rodents may have the greatest potential for uptake at the scales of individual residential properties or small clusters of neighboring properties, or, alternatively, spatially limited high-risk portions of public lands.

In the specific case of rodent-targeted acaricides, containment of the acaricide to devices should work in favor of homeowner acceptability of this approach. Surveys of homeowners in Lyme disease endemic areas indicate concern about the environmental impact and safety for pets and family members of synthetic acaricides when broadcast openly in the peridomestic environment (Gould et al., 2008; Beck et al., 2022). The same concern should not apply equally to rodent-targeted products based on synthetic acaricides that are contained to devices prior to being contacted by the rodents and designed to be delivered orally via a food source laced with acaricide and presented in a bait box (Poché et al., 2020, 2021); or topically via (i) treated cotton, for use as nesting material, presented in a tube (Mather et al., 1987, 1988), (ii) treated carpet strips lining the inside of a tube containing a food source (Gage et al., 1997; Lane et al., 1998), or (iii) a treated wick the animal brushes up against as it navigates toward a food source in a bait box (Dolan et al., 2004). Such delivery mechanisms minimize both environmental impact and the risk for non-target organisms, including pets and children, to come into contact with potentially harmful acaricide compounds. However, the public’s level of acceptability for different types of rodent-targeted approaches, including cost considerations, remains unclear.

Another general benefit of rodent-targeted acaricides is that they have the potential to directly impact tick infestation of rodents as well as indirectly impact pathogen infection in rodent reservoirs and questing vector ticks (Table 1). A successful intervention should result in reduction in the number of *Ix. scapularis* immatures (larvae and nymphs) completing their bloodmeal on target rodent species and reduction in infection prevalence in the rodent population, in turn leading to reduction in both the abundance of questing *Ix. scapularis* nymphs and adults and their prevalence of pathogen infection. The intervention thus impacts both factors (abundance of questing ticks and infection prevalence in questing ticks) contributing to the abundance of questing infected *Ix. scapularis* nymphs and adults, which is the gold standard acarological measure of human risk for infection (Eisen and Eisen, 2016).

### 2.2. General drawbacks

One main drawback of rodent-targeted approaches is that impact on acarological risk for human exposure to pathogen-infected *Ix. scapularis* nymphs, which are considered to account for the majority of pathogen transmission to humans by this species (Eisen and Eisen, 2016), is not expected until the year after the intervention is first put in place. A rodent-targeted intervention started in the spring of Year 0 is not expected to directly impact the abundance of questing infected *Ix. scapularis* nymphs in the spring or summer of Year 0, as these nymphs fed as larvae in the previous year. However, it could impact the abundance of questing infected *Ix. scapularis* adults in the fall of Year 0 because these adults result from nymphs fed in the spring or summer of Year 0. Should the intervention prove successful in Year 0, a reduction in the abundance of questing infected *Ix. scapularis* nymphs is expected to occur in Year 1. With one notable exception (see Section 3.2), all studies on topical application of acaricides to rodents (via permethrin-treated cotton or acaricides delivered via bait boxes) have viewed the abundance of questing *Ix. scapularis* nymphs in the spring of the year the intervention was started as pre-treatment data rather than being impacted by the intervention. Moreover, the 1-year lag time between implementation of a rodent-targeted approach and expected impact on acarological risk for human exposure to pathogen-infected *Ix. scapularis* nymphs may be part of why pest control firms use existing rodent-targeted tick control products sparingly when contracted by homeowners to control ticks on residential properties (Jordan and Schulze, 2020). As noted previously for use of rodent-targeted topical acaricide (Schulze et al., 2007), an additional tick control measure with immediate impact on questing nymphs, such as broadcast application of acaricide, is advisable to reduce the acarological risk for human tick encounters in the year when the rodent-targeted intervention is started.

Rodent-targeted approaches also have the inherent weakness of their success being dependent on (i) impacting a large proportion of the populations of target rodent species serving as pathogen reservoirs and (ii) non-target pathogen reservoirs, potentially including both rodent species not impacted by a given intervention and other vertebrate reservoir species, having a limited contribution to enzootic pathogen transmission. Both inadequate treatment rates for target rodent species and substantial contributions to production of infected fed larvae by non-target reservoir species can contribute to lack of impact on the prevalence of rodent-associated pathogens in questing *Ix. scapularis*, as recently reported by Hinckley et al. (2021) for rodent self-application of topical acaricide. Readily measured outcomes for a rodent-targeted intervention include the use of treatment delivery devices (e.g., by tracking the amount of food bait removed from treatment boxes or how much treated cotton was removed from tubes) and the abundance of pathogen-infected questing *Ix. scapularis* nymphs. However, should the intervention fail, these outcome measures are not sufficient to understand why it failed. Expanding the intervention evaluation to also include trapping and processing of target rodent species can provide additional information to help understand why an intervention succeeded or failed, such as clarifying the proportion of rodents that were exposed to the treatment, the level of tick infestation, and the infection status of the rodents. This, however, is work intensive and costly, especially for larger scale intervention studies.

The last piece of the puzzle is to determine the contribution to production of infected fed *Ix. scapularis* larvae by the target rodent species versus other pathogen reservoirs, including rodent species not making use of treatment delivery devices as well as shrews, medium-sized mammals, and birds (Mather et al., 1989; Fish and Daniels, 1990; Giardina et al., 2000; Brisson et al., 2008). Capturing and processing such a wide range of animal species to collect all information needed to estimate their respective contributions to production of fed infected larvae is simply not realistic across a large enough number of treatment and control sites in an intervention study. The alternative is to achieve this via collection and processing of questing ticks to determine not only their infection status but also identify the host species they fed on in the previous life stage. Based on the tremendous potential to improve our understanding of tick-host relationships as well as enzootic transmission cycles, many different identification techniques for blood remnants have been explored over the last two decades but identification of the host species that provided the larval blood meals for more than half of examined field collected questing nymphs has proven difficult (selected references include: Tobolewski et al., 1992; Kirstein and Gray, 1996; Pichon et al., 2003; Humair et al., 2007; Wickramasekara et al., 2008; Pierce et al., 2009; Schmidt et al., 2011; Gariepy et al., 2012; Scott et al., 2012; Önder et al., 2013; Collini et al., 2015; Hamer et al., 2015; LoGiudice et al., 2018; Heylen et al., 2019; Lumsden et al., 2021). Recent technological advances leading to increased detection sensitivity for partially degraded genetic material, combined with steadily improving bioinformatics databases for vertebrate animals, provide new opportunities for species identification of remnant blood in the majority of examined questing ticks (Goethert et al., 2021; Goethert and Telford, 2022a, 2022b). This could constitute a breakthrough not only for evaluating rodent-targeted interventions but also for assessing the likelihood of a rodent-targeted intervention to succeed based on determination of the contribution by the target rodent species, relative to all other pathogen reservoir species, to the production of fed infected larvae that molt into nymphs and then quest openly in the environment.

A final consideration is that while rodent-targeted approaches have strong potential to control *Ix. scapularis*, they are not effective against *Amblyomma americanum* (lone star tick) which can co-occur with *Ix. scapularis* on residential properties and in other settings presenting risk for human tick encounters in some parts of the eastern United States (Jordan and Egizi, 2019). The lack of impact on *Am. americanum* is due to this species not readily utilizing rodents as hosts (Zimmerman et al., 1987; Allan et al., 2010).

### 2.3. Unresolved issues

One issue yet to be resolved is to what extent food bait, used to attract rodents to treatment devices such as bait boxes, influences rodent microhabitat use and potential for population growth (Machtinger and Li, 2019), which in turn can impact abundance of questing ticks. The impact of food bait likely varies with fluctuations in access to natural food sources for rodents, for example caused by tree masting generating large crops of acorns or seeds (Ostfeld et al., 2006; Bregnard et al., 2020). Treatments to kill parasitizing ticks or eliminate pathogens from rodents also may have a positive impact on their health and reproductive potential, and thus could facilitate population growth. Two other issues having received only limited attention to date also should be considered in the future. Although rodent-targeted acaricides delivered via devices such as tubes or boxes have limited potential for being spread (via rodents and other animals) widely into the environment, there is the potential issue of predators consuming treated animals. There also is concern about antimicrobial resistance resulting from antibiotic treatment of rodent reservoirs to eliminate human pathogens (discussed further in Section 3.4).

## 3. Empirical evidence for impact of rodent-targeted approaches on outcomes associated with rodents, ticks, human tick encounters, and tick-borne disease

Field evaluations of rodent-targeted approaches commonly include outcome measures related to tick infestation of rodents, pathogen infection of rodents, abundance of questing ticks representing species potentially impacted by the intervention, and pathogen infection prevalence in the questing ticks (see Sections 3.1 to 3.7). Only two studies of rodent-targeted interventions published to date have additionally included data for human tick encounters and human tick-borne disease (Hinckley et al., 2021; Keesing et al., 2022); both studies examined self-application of topical acaricide by rodents (see Section 3.2).

### 3.1. Acaricide or entomopathogenic fungus delivered topically via treated cotton nesting material

The first field trial of this method was conducted from May to September 1985 in a Massachusetts woodland setting, with tubes containing permethrin-treated cotton deployed in late May at a density of approximately 82 tubes/ha, spaced 10 m apart in a grid pattern (Mather et al., 1987). Notable findings included (i) permethrin-treated cotton being removed from dispensing tubes, (ii) dramatic decreases in immature *Ix. scapularis* or *De. variabilis* infestations on *Pe. leucopus* from treatment sites compared with those from control sites (more than three-fold reduction in the proportion of infested mice, and the mean number of immature ticks collected per mouse reduced more than ten-fold), (iii) challenge of mice recovered from treatment and control sites with laboratory-reared *Ix. scapularis* larvae showing roughly six-fold reduction in larval feeding success for mice from treatment sites, (iv) no apparent impact of permethrin-treated cotton on mouse survival or reproduction, and (v) the intervention having no impact on ticks infesting *Microtus* voles, most likely because they did not make use of the treated cotton. As expected from the life cycle of *Ix. scapularis* in the Northeast, with nymphal peak activity in the spring preceding larval peak activity in the summer (Yuval and Spielman, 1990), there was no impact of the treatment from May to September 1985 on questing *Ix. scapularis* nymphs during the same time period.

Based on these promising results, a follow-up study was conducted from 1986 to 1987 in a residential setting in Massachusetts where tubes containing permethrin-treated cotton were deployed in May and August of both years, corresponding to the seasonal peaks for questing *Ix. scapularis* nymphs and larvae (Mather et al., 1988). Tubes were deployed 10 m apart in grids covering all potential mouse habitat on the participating residential properties. In addition to studying the impact of the intervention on *Ix. scapularis* immatures infesting *Pe. leucopus* over two years, there was an additional component in the spring of 1987 to assess the impact on abundance of questing *Ix. scapularis* nymphs and their prevalence of infection with *Bo. burgdorferi* s.l.; these nymphs were of the same cohort as larvae fed during the summer of 1986. The intervention resulted in near complete elimination of *Ix. scapularis* immatures on white-footed mice in 1986: a total of 3 larvae were recovered from 1 of 40 mice in the treatment sites, whereas 33 of 34 mice in the control sites were infested, harboring an average of 20 immatures. Moreover, in the spring of 1987, dramatic reductions in abundance of questing *Ix. scapularis* nymphs (ten-fold reduction compared to control sites) and prevalence of infection with *Bo. burgdorferi* s.l. in the nymphs (three-fold reduction compared to control sites) were observed in treatment sites compared to untreated control sites, combining for a 97% reduction in abundance of questing infected nymphs.

Following conditional registration by the Environmental Protection Agency (EPA) in 1987 and registration in 1988 (registration number, 56783–1), this tick control method was made commercially available under the product name DAMMINIX (also referred to as Damminix Tick Tubes^®^; Ecohealth, Inc., Boston, MA, USA). Soon thereafter, a suite of studies was conducted in residential areas and woodland settings in Connecticut, Massachusetts, and New York to evaluate this new tick control product (Daniels et al., 1991; Deblinger and Rimmer, 1991; Stafford, 1991, 1992; Ginsberg, 1992). The treatment scheme in these studies followed product label instructions, with tubes spaced roughly 10 m apart and deployed in spring against *Ix. scapularis* nymphs and in summer against larvae.

A study of Damminix Tick Tubes^®^ in a woodland setting in Massachusetts (Deblinger and Rimmer, 1991) reported reductions for infestation of *Pe. leucopus* by *Ix. scapularis* immatures (examined from 1987 to 1989) and abundance of questing *Ix. scapularis* nymphs in the spring (examined from 1989 to 1990) of similar magnitude as observed by Mather et al. (1988). However, other studies conducted in residential areas or woodland settings in the Northeast reported very different results using the same tube deployment scheme. Daniels et al. (1991) reported statistically significant reductions in the proportion of *Pe. leucopus* infested by *Ix. scapularis* larvae for Damminix Tick Tubes^®^ treatment sites, as compared to control sites, in two of three examined habitat types (residential and woodland) in the summer of 1988, but significant reduction in the level of infestation of individual *Pe. leucopus* by larvae was observed only for one habitat type (residential). Moreover, the three-fold level of reduction for number of larvae infesting mice in the residential setting was lower than the ten-fold level of reduction for immatures observed in this type of setting by Mather et al. (1988). Despite some impact on infestation of *Pe. leucopus* by *Ix. scapularis* larvae in 1988, there was no significant reduction for treatment sites in the following spring (1989) for either abundance of questing *Ix. scapularis* nymphs or prevalence of infection with *Bo. burgdorferi* s.l. in these nymphs in any of the examined habitat types. Similar results were reported from a Connecticut study conducted from 1989 to 1991 on Damminix Tick Tubes^®^ deployed in residential environments (Stafford, 1991, 1992). Despite more than ten-fold reductions in the mean number of *Ix. scapularis* larvae parasitizing *Pe. leucopus* in treatment sites in late summer of 1989 and 1990, there was no reduction in the spring of 1990 or 1991 for either abundance of questing *Ix. scapularis* nymphs or prevalence of infection with *Bo. burgdorferi* s.l. in these nymphs. Finally, Ginsberg (1992) reported variable outcomes after deployment of Damminix Tick Tubes^®^ on Fire Island, New York. Tick burdens were greatly reduced on *Pe. leucopus* in both sites examined but corresponding reductions in questing *Ix. scapularis* nymphs or their prevalence of infection with *Bo. burgdorferi* s.l. were not uniform across the sites. Factors speculated to have contributed to lack of impact on questing infected nymphs in some of the studies included a lower proportion of *Pe. leucopus* using the permethrin-treated cotton (perhaps due to greater access to natural nesting materials) and greater production of fed, infected larvae by non-*Peromyscus* tick hosts less willing, unwilling, or unable to access and use the permethrin-treated cotton as nesting material, such as *Microtus* voles, *Tamias* chipmunks, *Blarina* shrews, carnivores, or various species of birds.

Despite contradictory findings in these early studies, there were no additional published evaluations of the commercially available Damminix Tick Tubes^®^ until nearly three decades later, when Jordan and Schulze (2019) presented data for a comparison of two commercially available products for topical application of acaricide to rodents: Damminix Tick Tubes^®^ and SELECT TCS Tick Control System (Tick Box Technology Corporation, Norwalk, CT, USA). Both products were deployed over two years (2014 and 2015), in spring against *Ix. scapularis* nymphs and in summer against larvae. Treatment sites were residential settings for both products, whereas the control site was a natural area. It also should be noted that Damminix Tick Tubes^®^ were deployed at a lower density (20 m spacing between tubes) than specified on the product label (9.1 m or 10 yd spacing between tubes), whereas the SELECT TCS label is more flexible with regards to deployment density (a minimum of 10 m apart). This study presented data for two different rodent species serving as hosts for *Ix. scapularis* immatures and reservoirs for *Bo. burgdorferi* s.s.: *Pe. leucopus*, the main target for Damminix Tick Tubes^®^, and *Tamias striatus* (eastern chipmunk), which is not expected to make extensive use of the permethrin-treated cotton as nesting material. Data interpretation was complicated by low numbers of animals examined across treatments, years, and sampling months, especially for *Ta. striatus* with very few animals trapped in the woodland control site. For *Pe. leucopus*, reductions in tick burden following deployment of Damminix Tick Tubes^®^ on residential properties, compared to the control site, were reported for *Ix. scapularis* nymphs in both 2014 and 2015 (two-fold reduction in mean number of nymphs per mouse) but only in the second year for larvae (four-fold reduction). Unfortunately, there was no significant reduction in abundance of questing *Ix. scapularis* nymphs in the spring of either 2015 or 2016 in the Damminix Tick Tubes^®^ treatment sites. Results for SELECT TCS bait boxes are presented in Section 3.2. It remains unclear to what extent deploying Damminix tubes at half the label-recommended density may have impacted the results. Jordan and Schulze (2019) also noted that, compared to SELECT TCS, Damminix Tick Tubes^®^ holds advantages in that they can be purchased and deployed by homeowners as well as being roughly ten times less costly to apply (annual estimated cost of approximately $150 for 20 m spacing or $300 for 10 m spacing of Damminix Tick Tubes^®^ for a 1 ha property based on two applications, in spring and summer).

Three additional recent studies merit mention here. Mandli et al. (2021) examined the impact of a do-it-yourself (DIY) version of the permethrin-treated cotton intervention (PVC pipe containing cotton soaked in a water-based solution made from permethrin concentrate and then dried) on *Ix. scapularis* over five years (2014 to 2018) in a Wisconsin woodland setting, both as a single intervention and combined with removal of invasive vegetation. Deployment density followed that recommended for Damminix Tick Tubes^®^, with the DIY devices spaced 10 m apart. The study monitored cotton removal and assessed abundance of questing *Ix. scapularis* nymphs and their prevalence of infection with *Bo. burgdorferi* s.s. but did not evaluate tick infestation on rodents. In most years, the vast majority of treated cotton was removed from the PVC pipes during both spring and summer deployments. When used as a single control method, the DIY tube with permethrin-treated cotton resulted in 53% reduction in abundance of questing *Ix. scapularis* nymphs, and 66% reduction in abundance of *Bo. burgdorferi* s.s.-infected nymphs, in the following spring and early summer (across the 2015–2017 period when an impact was expected for questing nymphs). As the study did not include small mammal trapping, the successful outcome unfortunately cannot be placed in the context of the composition of the local rodent community. Brown et al. (2020) conducted a single-year study in a Pennsylvania woodland setting where Thermacell tick control tubes (Thermacell Repellents, Inc., Bedford, MA; EPA registration number 71910–10) were deployed in summer (August) to deliver permethrin-impregnated cotton to rodents, with tubes spaced 10 m apart. There was a complete lack of infestation of *Pe. leucopus* by *Ix. scapularis* immatures in the month (September) following treatment, whereas mice in control sites remained infested, but it was not determined if the treatment also resulted in a reduction of questing *Ix. scapularis* nymphs in the spring of the following year. Green et al. (2022) explored the impact of the month of placement for Damminix Tick Tubes^®^ and Thermacell tick control tubes, and modifications to the cotton nesting material, on utilization by rodents. Removal of cotton was monitored in the field (Pennsylvania) from July to October 2020, with peak cotton utilization recorded for October. Neither the size of cotton balls nor addition of odor attractants (peanut butter odor, vanillin odor, or safflower oil odor) were found to significantly impact mouse visits to the tubes or cotton removal.

Three older studies also should be mentioned. Leprince and Lane (1996) provided permethrin-impregnated cotton to *Neotoma fuscipes* (dusky-footed wood rat) via metal cylinders placed adjacent to their nests (houses) in a California chaparral brush setting. The intervention resulted in reduced infestation of *Ne. fuscipes* by *Ix. pacificus* larvae but not by immatures of the Pacific Coast tick, *Dermacentor occidentalis*. Culture of ear biopsies from *Ne. fuscipes* for *Borrelia* spirochetes indicated no difference in infection prevalence in treatment and control sites. Uptake of the treated material by *Ne. fuscipes* was limited, with cotton found in only 25% of examined woodrat nests; the authors speculated that most of the cotton disappearing from the metal cylinder had been removed by other rodents, such as *Peromyscus* mice. In a woodland setting in Sweden, Mejlon et al. (1995) explored the use of Damminix Tick Tubes^®^, together with tubes containing permethrin-impregnated paper, against *Ixodes ricinus* (the castor bean tick). The mean number of *Ix. ricinus* larvae infesting rodents in treatment sites was significantly reduced for *Myodes glareolus* (bank vole; formerly *Clethrionomys glareolus*) but not for *Apodemus flavicollis* (yellow-necked field mouse). Moreover, the abundance of questing *Borrelia*-infected *Ix. ricinus* nymphs was significantly reduced in treatment sites, compared to control sites, but only following two years of delivery of permethrin-impregnated cotton and paper. Finally, Hornbostel et al. (2005) offered *Pe. leucopus* nesting material in the form of cotton treated with the entomopathogenic fungus *Metarhizium anisopliae* (rather than permethrin) in a New York State woodland setting. An initial laboratory trial showed 75% mortality for *Ix. scapularis* larvae fed on mice using *Me. anisopliae-*treated nesting material, as compared with 35% for control mice. However, the subsequent field trial found no significant impact of *Me. anisopliae-*treated cotton, presented via nest boxes affixed to tree trunks, on the numbers of *Ix. scapularis* immatures infesting *Pe. leucopus*, or on the abundance of questing *Ix. scapularis* nymphs or the prevalence of infection with *Bo. burgdorferi* s.l. in the nymphs in the year following treatment.

Modeling approaches have been used to explore the impact of Damminix Tick Tubes^®^ on the abundance of questing *Ix. scapularis* nymphs infected with *Bo. burgdorferi* s.s. (Mount et al., 1997) and human Lyme disease cases (Hayes et al., 1999). Not surprisingly, the intervention was found to be sensitive to the coverage (% individuals treated) of target rodent species as well as the presence of non-targeted *Bo. burgdorferi* s.s. reservoirs. Assuming per-hectare host densities of 15 *Pe. leucopus*, 10 other small mammals and birds, 1.5 medium-sized mammals, and 0.25 deer, it was estimated that 99% of the mice within the intervention area must be treated to reduce the abundance of infected nymphs by 67% in year 3 and 78% in year 5 (Mount et al., 1997). Moreover, treatment of 90% of the *Pe. leucopus* was estimated to result in no more than 56% reduction in the abundance of infected nymphs even after 10 years of intervention. Finally, Hayes et al. (1999) found estimates for reducing Lyme disease cases to vary more than 100-fold based on variation in the proportion of a model community treated, the percentage of mice present that were successfully treated, and the level of tick mortality on treated mice.

### 3.2. Acaricide delivered topically via animal movement through a bait box

Compared to offering treated nesting material, using a food bait to entice animals to come into contact with topically applied acaricide has the potential to attract a wider range of rodent species. Early food-baited devices were designed to force the animals to navigate through: (i) a rectangular box where they had to pass through a set of felt-covered wheels treated with acaricide to access the food bait (Sonenshine and Haines, 1985); or (ii) an open PVC cylinder where they had to pass acaricide-treated textile strips, glued to the inside of both ends of the cylinder, in order to reach a food bait in the middle of the cylinder (Sonenshine and Haines, 1985; Gage et al., 1997; Lane et al., 1998). Field trials showed that use of these devices could reduce infestation levels by 80–100% for *De. variabilis* immatures on *Pe. leucopus* and *Microtus pennsylvanicus* (meadow vole) in Virginia (Sonenshine and Haines, 1985), *Ixodes spinipalpis* on *Neotoma mexicana* (Mexican wood rat) in Colorado (Gage et al., 1997), and *De. occidentalis* and *Ix. pacificus* on *Ne. fuscipes* in California (Lane et al., 1998).

Subsequent efforts focused on development and commercialization of a new device for topical application of acaricide to control *Ix. scapularis* immatures on *Peromyscus* mice as well as *Tamias* chipmunks (Dolan et al., 2004). Both types of rodents are abundant in residential settings in the Northeast, heavily infested by *Ix. scapularis* immatures, and effective reservoirs for *Bo. burgdorferi* s.s. (Levine et al., 1985; McLean et al., 1993; Schmidt et al., 1999; Schulze et al., 2005). An approach impacting both *Peromyscus* mice and *Tamias* chipmunks was therefore theorized to be more robust than delivery of permethrin-treated cotton as nesting material, which primarily targets *Peromyscus* mice. The new device consists of a bait box where animals are treated topically with acaricide (fipronil) on their back via a wick as they are navigating toward a food bait. It was initially marketed in 2004 under the brand names Maxforce Tick Management System^™^ (TMS) or Select TCS Rodent Station (Bayer CropScience LP, Montvale, NJ, USA; EPA registration number 432–1248), but since 2012 has been marketed under the brand names SELECT TCS Tick Control System and TICK BOX^™^ Tick Control System (Tick Box Technology Corporation, Norwalk, CT, USA; EPA registration number 85306–1). As currently approved for use nationally by the EPA, SELECT TCS Tick Control System and TICK BOX^™^ Tick Control System can only be deployed by pesticide management professionals or public health department personnel (they are not available as over-the-counter products for use by homeowners). Individual states may have additional regulations. The device includes a child-resistant rodent bait box and a metal shroud to prevent damage by non-target animals trying to access the food bait (especially *Sciurus* tree squirrels; (see Schulze et al. 2007, Dolan et al. 2017). These requirements have led to a high cost for use of this product, with an estimated retail cost of $50 per SELECT TCS unit (including the box, metal cover, anchoring stakes, deployment and retrieval labor, and disposal) and an estimated annual total cost of more than $2000 to treat the wooded portion of a 1 ha property based on 20 m spacing between devices and two deployments per year, one in spring and one in summer (Jordan and Schulze, 2019; T.L. Schulze, personal communication regarding current cost per SELECT TCS unit). Using the manufacturer minimum recommended 10 m spacing distance between boxes, this cost would double.

Maxforce TMS and SELECT TCS have been evaluated both as a single tick and pathogen control intervention (Dolan et al., 2004; Schulze et al., 2017; Jordan and Schulze, 2019; Hinckley et al., 2021; Keesing et al., 2022) and as part of an integrated tick/pathogen management approach in combination with (i) broadcast of entomopathogenic fungus (Williams et al., 2018a, 2018b; Little et al., 2020; Keesing et al., 2022; Linske et al., 2022), (ii) deer population reduction and broadcast of entomopathogenic fungus (Williams et al., 2018a, 2018b; Little et al., 2020, Linske et al., 2022), (iii) topical acaricide treatment of deer and broadcast of synthetic acaricide (Schulze et al., 2007), and (iv) antibiotic treatment of rodents (Dolan et al., 2017). Studies evaluating Maxforce TMS or SELECT TCS as a single control method have produced variable results for key acarological outcome measures and the reason for this is difficult to elucidate as the studies have had variable study designs, including for density of deployed bait boxes, and included different suites of outcome measures (study characteristics are summarized in Table 2).

The initial field study was conducted in Connecticut from 1999 to 2001 and explored several different bait box prototypes, including use of different fipronil-treated materials to treat the rodents topically (Dolan et al., 2004). The treatment arm of the study was robust, including approximately 150 residential properties, but the control sites were located in natural woodland rather than representing residential properties. Boxes were frequently used by rodents, as evidenced by removal of bait, and results for acarological outcomes in treatment versus control sites were promising with reductions in treatment sites for numbers of *Ix. scapularis* infesting *Pe. leucopus* (84% reduction for larvae and 68% for nymphs), prevalence of *Bo. burgdorferi* s.l. infection in the mice (53% reduction), abundance of questing *Ix. scapularis* nymphs (62 to 97% reduction), prevalence of *Bo. burgdorferi* s.l. infection in the nymphs (60% reduction), and abundance of infected host-seeking nymphs (85% reduction) (see Table 6 of Eisen and Dolan 2016). However, it should be noted that bait was periodically replaced in the boxes as needed to promote continual use.

Following commercialization of Maxforce TMS, this product was used in two studies conducted in residential settings in Monmouth County, New Jersey from 2003 to 2009 (Schulze et al., 2007; Dolan et al., 2017). Neither of these studies used Maxforce TMS as a single control method but some findings nevertheless are of interest. Schulze et al. (2007) deployed Maxforce TMS with three different spacing intervals for boxes placed along ecotones or in parallel rows in wooded habitat: 10, 15, and 20 m. There were no clear differences among these spacing distances for either box use or infestation of *Pe. leucopus* and *Ta. striatus* by *Ix. scapularis* immatures. Dolan et al. (2017) used Maxforce TMS enhanced with bait laced with antibiotic (doxycycline hyclate); consequently, as noted in Table 2, only the results for tick infestation of rodents and abundance of questing ticks can be attributed to the topical acaricide treatment (and see Section 3.4 for results relating to pathogen infection). In this case, Maxforce TMS boxes were deployed spaced 25 m apart in two rows along lawn ecotone with woods and 10 m into wooded habitat. Reductions were recorded in treatment sites for infestation of *Pe. leucopus* and *Ta. striatus* by *Ix. scapularis* immatures (65 to 94% reduction) and abundance of questing *Ix. scapularis* nymphs (68 to 77% reduction). As in the previous study by Dolan et al. (2004), bait was periodically replaced in the boxes as needed to promote continual use.

Two subsequent studies evaluated SELECT TCS as a single control method in Monmouth County and Ocean County, New Jersey, with treatments conducted over a 2-yr period from 2012 to 2013 (Schulze et al., 2017) or 2014 or 2015 (Jordan and Schulze, 2019). Both were small scale studies with 6 to 12 treated residential properties and a natural area serving as the control. Boxes were not rebaited during these studies; to mimic operational use, boxes deployed in spring against *Ix. scapularis* nymphs were simply replaced with new boxes approximately two months later in summer, targeting infestation by *Ix. scapularis* larvae. Outcome measures presented in the publications included bait box use, infestation of rodents by *Ix. scapularis* immatures, and abundance of questing *Ix. scapularis* nymphs, but not pathogen infection in rodents or questing ticks. The studies differed in terms of both box density (boxes spaced 10 versus 20 m apart) and box placement on the properties (rows located 3 and 13 m into woods from lawn ecotone versus 10 and 30 m into woods from lawn ecotone) (see Table 2). Regardless of these differences, both studies indicated dramatic reductions in residential treatment sites, compared to the natural control area, for infestation of *Pe. leucopus* and *Ta. striatus* by *Ix. scapularis* larvae in the summer (in most cases five-fold or greater reduction in numbers of infesting larvae) and abundance of questing *Ix. scapularis* nymphs of the same cohort in the spring of the following years (79 to 97% across studies and years). The impact on infestation of rodents by *Ix. scapularis* nymphs in the spring was mixed across studies and years (with reductions for the number of nymphs infesting rodents in only half of the cases), potentially due to the treatment commencing in May and nymphal infestation being assessed in June (roughly 1 month after bait box deployment) rather than in July or August as for larval infestation (after 2–3 months of bait box deployment). As noted previously in Section 3.1, the second study (Jordan and Schulze, 2019) included a comparison of SELECT TCS and Damminix Tick Tubes^®^.

Up to this point, all studies on Maxforce TMS/SELECT TCS had shown promising results, with reductions in treatment sites for: (i) infestation by *Ix. scapularis* of rodents serving as important hosts for immature ticks as well as pathogen reservoirs; and (ii) abundance of questing *Ix. scapularis* nymphs, which are considered the primary vectors to humans of the causative agents of Lyme disease, anaplasmosis, and babesiosis. The initial Connecticut study on the product prototype (Dolan et al., 2004) also recorded reductions for prevalence of infection with *Bo. burgdorferi* s.l. in *Pe. leucopus* as well as questing *Ix. scapularis* nymphs, but these outcome measures were not included in the publications for subsequent New Jersey studies on SELECT TCS as a single control method (Schulze et al., 2017; Jordan and Schulze, 2019). Moreover, data presented from New Jersey (Schulze et al., 2007,^2017^; Dolan et al., 2017; Jordan and Schulze, 2019) also indicated similar results for Maxforce TMS/SELECT TCS when deployed at variable box density. This is an important point as the high cost of SELECT TCS would tend to drive residential deployments toward using the least costly label-recommended application scheme, with boxes placed near the edge of maintained landscaping and woodlots and/or brush, rather than also adding a second row of boxes ~10 m into the wooded portion of residential properties containing such habitat. Extending the box spacing within rows from the label-recommended minimum distance of 10 m to 20 m could allow for including a second row of boxes within wooded habitat without increasing the cost.

As this promising control approach has potential to suppress questing *Ix. scapularis* across all tick habitat on a residential property, there was interest in evaluating the impact also for human bites by this tick species and its associated tick-borne diseases. This was done in two multi-year studies conducted in Connecticut (2012–2016; Hinckley et al., 2021) and New York (2017–2020; Keesing et al., 2022) with both treatment and control arms represented by residential properties, randomly assigned, and robust sample sizes for numbers of included properties (~220 to 270 per study arm) and human participants (~560 to 1000 per study arm) (see Table 2 for summary of study characteristics). The Connecticut study followed the least costly label-recommended scheme for deployment of SELECT TCS: boxes spaced 10 m apart in a row located 3 m into woods or brush from ecotone with lawn and with boxes also placed along unsealed stone walls and wood piles (the average property included 10 bait box locations). The New York study described their deployment of SELECT TCS as boxes being placed ≥10 m apart in all habitat types sampled for ticks (lawn, forest, and shrub/garden), with placement in protected locations, such as along building foundations and under vegetation, frequently used by small mammals (the average property included 6 bait box locations; ~38 boxes/ha). Boxes were not rebaited in either study; to mimic operational use, boxes deployed in spring against *Ix. scapularis* nymphs were simply replaced with new boxes approximately two months later in summer, targeting infestation by *Ix. scapularis* larvae. Neither study provided an estimate for the cost of the SELECT TCS treatment of residential properties, although based on an average number of 10 units placed along wooded ecotones per property in the Connecticut study, and deployments in both spring and summer, the annual cost would have been in the range of $1000 per property ($50 per unit × 20 units per year). Extending the treatment to also include SELECT TCS units placed in the wooded portion of a property likely would have doubled the annual cost.

The outcome measures included in the publications describing these studies differed notably. The Connecticut study did not include outcome measures associated with rodents, whereas the New York study presented data for infestation by ticks of *Pe. leucopus* and *Ta. striatus* (the vast majority of these ticks were *Ix. scapularis* immatures; F. Keesing, Bard College, NY, USA, personal communication). Moreover, the Connecticut study presented data for both abundance of questing *Ix. scapularis* nymphs and their prevalence of infection with human pathogens whereas the New York study presented data only for abundance of questing nymphs (99% were *Ix. scapularis* nymphs; F. Keesing, personal communication). Both studies presented data for human tick encounters, but neither study presented data broken down by tick species (see Eisen 2022 for a discussion of why the lack of this information is unfortunate). Data for self-reported tick-borne disease of humans was reported in both studies. Finally, it should be noted that the New York study also presented data for pet encounters with ticks and tick-borne disease of pets.

Overall, the findings were disappointing in both studies. Treatment of residential properties in the Connecticut study failed to significantly reduce any of the following outcome measures when compared to the control properties: abundance of questing *Ix. scapularis* nymphs; prevalence of infection in the nymphs with *Bo. burgdorferi* s.s.; household level reports of ticks found crawling on or biting residents; or household level reports of self-reported tick-borne disease (Hinckley et al., 2021). Reasons remain unclear for the unexpected failure to significantly impact questing *Ix. scapularis* nymphs, in contrast to previous small-scale studies on SELECT TCS in New Jersey (Schulze et al., 2017; Jordan and Schulze, 2019) and forested habitat in the large-scale study in New York (Keesing et al., 2022). As tick infestation and pathogen infection of target rodent species were not assessed in the Connecticut study, it is not clear to what extent the failure to suppress questing ticks or reduce their pathogen infection prevalence was caused by inadequate treatment of the populations of target rodent species (for example via a very high ratio of rodent individuals to boxes) versus strong contributions to feeding of *Ix. scapularis* larvae by host species not impacted by the control approach, such as shrews, tree squirrels, or birds.

One main difference between the Connecticut study and the previous New Jersey studies on SELECT TCS was that the latter studies deployed boxes not only along the woods-lawn ecotone but also within the wooded habitat, thus potentially treating a larger proportion of the rodents present on the properties. However, in this context it is also worth noting that Linske et al. (2022) explored deployment of boxes in different configurations as part of integrated tick management evaluations conducted in Connecticut from 2013 to 2016 and including SELECT TCS (Williams et al., 2018a, 2018b; Little et al., 2020). When using a fixed number of boxes (*n* = 10), placement along the woods-lawn ecotone (~2 m into the woods) with a 10 m spacing between boxes was found to be more effective in reducing burdens of *Ix. scapularis* immatures on *Pe. leucopus* compared to placement in a grid (2 × 5 configuration at 10 m spacing between boxes) extending from the ecotone into the wooded portion of the properties but only covering the middle section of the backyard rather than the entire ecotone. Moreover, a multiple linear regression analysis revealed that box deployment configuration and total *Pe. leucopus* captures per trap night were significant predictors of burden of *Ix. scapularis* immatures on *Pe. leucopus*, whereas average bait consumption was not a significant predictor. Intriguingly, tick burdens decreased with increasing *Pe. leucopus* captures per trap night. Another recent study exploring use of SELECT TCS bait boxes by *Pe. leucopus* underscored the value of flexible placement of individual boxes in microhabitats that are heavily used by the target species, and also raised the issue of how the food bait may impact mouse population dynamics (Machtinger and Li, 2019). Additional efforts are merited to better understand how SELECT TCS boxes need to be deployed to maximize the impact on *Ix. scapularis* immatures infesting *Pe. leucopus* and *Ta. striatus* while at the same time keeping implementation cost down for homeowners. One specific topic to explore is how the efficacy of SELECT TCS in treating rodents (determined by detection of fipronil in blood from captured rodents) and reducing their tick burdens changes as the ratio of rodent individuals to deployed boxes increases, taking into consideration seasonal and inter-annual variation in natural food sources. For example, Schulze et al. (2017) noted that the percentage of boxes emptied of bait during a 9-wk summer deployment (following the SELECT TCS product label, which recommends switching out boxes 75–90 d after deployment) increased from 15% after 2 wk to 46% after 4 wk and 81% when the boxes were removed at the end of the 9-wk deployment. Boxes being emptied part way through their deployment could be countered by more frequent box replacement or re-baiting of boxes, but this would incur considerable cost for the homeowner paying for service provided by pesticide management professionals.

The other large-scale evaluation of SELECT TCS as a single control method, conducted in New York, generated mixed results (Keesing et al., 2022). Treatment properties had a statistically significant roughly 50% reduction in mean number of ticks infesting *Pe. leucopus* (the vast majority of these ticks were *Ix. scapularis* immatures; F. Keesing, personal communication) but there was no similar reduction for *Ta. striatus*. There also was a statistically significant 53% reduction in the abundance of questing nymphs (99% were *Ix. scapularis* nymphs; F. Keesing, personal communication) in forested habitats on the treated properties, based on a neighborhood level analysis. However, no similar reduction was seen for either per-capita human encounters with ticks (the majority of these ticks most likely were *Ix. scapularis*; F. Keesing, personal communication) or self-reported human tick-borne disease. It is also worth mentioning that the intervention evaluation mentioned above was part of a larger study also including an arm that combined broadcast application of an entomopathogenic fungus product (Met52; Novozymes Biologicals, Inc., Salem, VA, USA) with deployment of SELECT TCS. Keesing et al. (2022) noted in the Methods section that: “If effective, TCS bait boxes would kill larval (hatchling stage) ticks feeding on small-mammal hosts in summer and fall, leading to fewer nymphs (second immature stage) the following spring. Met52 would kill questing nymphal ticks in spring.”

A final observation on the study by Keesing et al. (2022) is that data for questing nymphs from the first intervention year with SELECT TCS are treated as having been impacted by the intervention in the statistical analyses. This could not have resulted from impact on the larval stage of the same tick cohort, which fed in the summer of the year before the intervention started. Any potential impact on questing nymphs in the year the intervention started therefore must have resulted from nymph-rodent interactions in the spring of the first intervention year, which differs from subsequent intervention years when there also was an impact on the preceding larval stage of the same tick cohort. As noted previously, all other studies on topical application of acaricides to rodents (via permethrin-treated cotton or acaricides delivered via bait boxes) have viewed the abundance of questing *Ix. scapularis* nymphs in the spring of the year the intervention was started as pre-treatment data rather than being impacted by the intervention.

### 3.3. Orally delivered acaricide

Orally delivered acaricides (isoxazolines) to kill feeding ticks are commonly used in dogs (Stafford, 2017). However, orally delivered acaricides are currently not commercially available for use to kill ticks feeding on wild rodents. Benefits to an orally delivered acaricide include potential for treatment of a wider range of small mammals compared to permethrin-treated cotton in Damminix Tick Tubes^®^ and a more direct treatment mechanism compared to the fipronil-impregnated wick for topical application in SELECT TCS. There also is the intriguing possibility of a downstream rodent bait pellet treated with a combination of acaricide and vaccine against *Bo. burgdorferi* s.s.

Studies on orally delivered acaricides to kill ticks infesting rodents are rare. An older study from California found that bait laced with an arthropod development-inhibitor (fluazuron) failed to control ticks, including *De. occidentalis* and *Ix. pacificus*, on *Ne. fuscipes*, whereas it was effective against fleas (Slowik et al., 2001). A previous laboratory study by Gray et al. (1994) showed that oral treatment with fluazuron reduced the molting success for *Ix. ricinus* larvae or nymphs having fed on treated Mongolian gerbils (*Meriones unguiculatus*), and the ticks that did molt were unable to attach and feed in the subsequent nymphal or adult stages. Recent studies have explored the use of two other acaricides – fipronil and the isoxazoline fluralaner – to control *Ix. scapularis* on *Peromyscus* mice. Pelletier et al. (2020) reported that fluralaner (administered at 12.5 or 50 mg/kg) ingested orally by *Peromyscus maniculatus* (eastern deer mouse) in the laboratory on a single occasion killed >90% of larvae feeding on mice 2 d post-treatment (based on numbers of larvae attached and still living 48 h after tick introduction) but there was no reduction in feeding success for larvae placed on mice 28 or 45 d post-treatment. This was likely related to the concentration of fluralaner in mouse blood decreasing more than 10-fold from day 2 to 28 post-treatment. A subsequent multi-year field study (Pelletier et al., 2022) evaluated treatment with a bait consisting of a mixture of peanut butter and the commercial formulation of fluralaner, Bravecto (Merck Animal Health, Madison, NJ, USA). The bait was delivered from July to August via Protecta RTU bait stations (Bell Laboratories, Inc., Madison, WI, USA) at two different densities: 2.1 and 4.4. bait stations per 1000 m^2^. Bait was replenished weekly. The average number of *Ix. scapularis* infesting *Peromyscus* mice in treatment sites, compared to control sites, was reduced by 68% for larvae but not reduced for nymphs with the lower bait station density, whereas it was reduced by 86% for larvae and 72% for nymphs with the higher bait station density. Abundance of questing ticks was not included as an outcome measure for the intervention.

Poché et al. (2020, 2021) conducted similar work in laboratory and simulated field settings for oral treatment of *Pe. leucopus* with fipronil against *Ix. scapularis*. Treatment was in the form of formulated rodent bait containing 0.005% fipronil in both studies. In the laboratory study (Poché et al., 2020), mice were fed treated rodent bait over a 48-h period and then challenged with *Ix. scapularis* larvae 1, 9 and 15 d post-treatment. Multiple outcome measures relating to attachment and feeding success that spanned the entire period of larval feeding were recorded. The treatment provided 100% control of *Ix. scapularis* larvae (no introduced larvae were able to attach, feed to completion and then detach) up to 15 d post-treatment. In a subsequent simulated field study (Poché et al., 2021), *Pe. leucopus* were held in enclosures and allowed to choose between fipronil-treated rodent bait and an alternative food source (consisting of equal parts commercial rodent diet and rolled oats) recommended by EPA for use in choice tests involving *Peromyscus* mice. Mice offered fipronil-treated rodent bait over a 24-h period were challenged with *Ix. scapularis* larvae at 1 and 15 d post-treatment, whereas mice offered fipronil-treated rodent bait over an extended 1-wk period were challenged with larvae 21 and 35 d post-treatment. Outcomes included measures relating to attachment and feeding success as well as molting success, which could be impacted by fipronil exposures that were sublethal during the larval feeding period. For a combined measure accounting for both success of attached larvae in feeding to repletion and to molt into nymphs, the 24 h treatment period resulted in 100% control 1 d post-treatment and 91% control 15 d post-treatment, whereas the 1-wk treatment period resulted in 92% control 21 d post--treatment and 82% control 35 d post-treatment. Analysis of the concentration of fipronil sulfone (a fipronil metabolite) for individual mice revealed that the vast majority of replete larvae came from treatment group mice with undetectable plasma levels of fipronil sulfone, potentially representing individuals not favoring the fipronil-based rodent bait over the competing diet. Based on these promising results, the formulated fipronil-treated rodent bait is now undergoing a field trial in a Lyme disease endemic area of the northeastern United States, and efforts are underway to register the formulated fipronil-treated bait as a commercial product to control ticks on wild rodents (D. Poché, Genesis Laboratories, Inc., Wellington, CO, USA; personal communication).

### 3.4. Orally delivered antibiotic

The potential for oral antibiotic (doxycycline hyclate) treatment of rodents, via gavage, to prevent infection following recent exposure to *Ix. scapularis* nymphs infected with *Bo. burgdorferi* s.s. or *An. phagocytophilum* was first explored in *Mus musculus* with limited success (Zeidner et al., 2004, 2008; Massung et al., 2005). However, subsequent oral treatment of *Mu. musculus* mice by means of a doxycycline hyclate-laced rodent bait formulation demonstrated both protection of naïve mice against challenge with *Bo. burgdorferi* s.s.-infected *Ix. scapularis* nymphs (protection was afforded by the mice consuming treated bait over the 4-d duration of nymphal attachment) and cure of established infection caused by tick bite (Dolan et al., 2008). This was followed up with two field studies conducted in Monmouth County, New Jersey, to determine the potential for antibiotic treatment of natural rodent reservoirs to disrupt enzootic transmission of *Bo. burgdorferi* s.s. and *An. phagocytophilum* (Dolan et al., 2011, 2017).

The initial field study (Dolan et al., 2011) was conducted from 2007 to 2009 in a New Jersey mixed residential and woodland setting where doxycycline hyclate-laden bait was delivered to rodents via bait stations (Protecta LP; Bell Laboratories, Inc.). Bait stations were deployed from May to September of 2007 and 2008 in wooded habitat, with ~20 m spacing between stations along two concentric perimeters at distances of ~5 and 25 m from the lawn-woods ecotone. The stations were examined regularly and rebaited as needed during the deployment. Key outcome measures included infection with *Bo. burgdorferi* s.s. and *An. phagocytophilum* in: (i) small mammals (primarily *Pe. leucopus* and *Ta. striatus*) captured pre-intervention (May of 2007 and 2008) and post-intervention (June and August of 2007 and 2008); and (ii) questing *Ix. scapularis* nymphs collected pre-intervention (spring of 2007) and post-intervention (June of 2008 and 2009). Compared to control sites and pre-intervention data from treatment sites, the intervention resulted in very strong suppression of *Bo. burgdorferi* s.s. in small mammals and questing *Ix. scapularis* nymphs (no infection detected in 102 examined animals, and ten-fold reduction in infection prevalence for questing nymphs), and strong suppression also for *An. phagocytophilum*. A second study (Dolan et al., 2017) was conducted with rodent treatment from July to September 2008 and May to September 2009. The overall design was similar to the previous study (Dolan et al., 2011) but in this case the doxycycline hyclate-laden bait was presented in Maxforce TMS boxes equipped with fipronil-treated wicks for topical application to rodents. The sole outcome in this study that can be attributed exclusively to the doxycycline hyclate treatment is an observed reduction, compared to control sites and May-June 2008 pre-intervention data from treatment sites, for infection of small mammals with *Bo. burgdorferi* s.s. and *An. phagocytophilum* in August of 2008, with more than ten-fold reduction in infection prevalence for both pathogens. Additional presented outcomes for pathogen infection in small mammals or questing *Ix. scapularis* likely were influenced both by the doxycycline hyclate treatment and fipronil-based killing of immature ticks feeding on key rodent pathogen reservoirs.

Dolan et al. (2011) clearly recognized that there are concerns about the potential for development of microbial resistance after long-term use of a frontline broad-spectrum antibiotic, such as doxycycline, to treat rodents in the field. They noted that downstream studies could explore the use of alternative antibiotics. A decade later, Leimer et al. (2021) identified hygromycin A (produced by *Streptomyces hygroscopicus*) as an antibiotic that is highly selective against spirochetes, including *Bo. burgdorferi* s.l. Moreover, laboratory experiments with rodents showed that hygromycin A given via oral gavage cleared infection with *Bo. burgdorferi* s.s. from *Mu. musculus* and *Pe. leucopus*, and that hygromycin A presented in a rodent bait formulation cleared infection with *Bo. burgdorferi* s.s. from *Mu. musculus* as effectively as doxycycline (Leimer et al., 2021). Based on these promising results, a formulated hygromycin A-treated rodent bait is now undergoing a field trial in a Lyme disease endemic area of the northeastern United States (S.R. Telford, III, Tufts University, Medford, MA, USA; personal communication)

### 3.5. Orally delivered anti-tick vaccine

Development of anti-tick vaccines against human biting ticks have focused primarily on protection of humans against tick-borne infection via pathogen transmission-blocking anti-tick vaccines (see reviews by Rego et al. 2019, van Oosterwijk 2021, van Oosterwijk and Wikel 2021). However, some anti-tick vaccine candidates, including subolesin and salivary proteins, have been shown to disrupt feeding by *Ix. scapularis* immatures on immunized guinea pigs (*Cavia porcellus*) or *Mu. musculus*, and to reduce transmission of *Bo. burgdorferi* s.s. or *An. phagocytophilum* at the tick-rodent interface (Almazán et al., 2005; de la Fuente et al., 2006; Dai et al., 2009; Bensaci et al., 2012; Narasimhan et al., 2020; Sajid et al., 2021). As noted by van Oosterwijk and Wikel (2021), such anti-tick vaccine candidates could be of interest for use in natural rodent reservoirs to reduce tick feeding success and intensity of enzootic pathogen transmission. I am not aware of any publications on the topic of developing an oral anti-tick vaccine bait for wild rodents with the specific goal of disrupting feeding by *Ix. scapularis* immatures.

### 3.6. Orally delivered vaccine against Bo. burgdorferi s.s

Seminal work by Kurtenbach et al. (1997) in Europe on experimental immunization of a natural rodent reservoir (*Ap. flavicollis*) using recombinant lipidated outer surface protein A (OspA) from *Bo. burgdorferi* s.s. spurred interest in the concept of vaccinating rodent reservoirs against Lyme disease spirochetes to disrupt enzootic transmission cycles. Soon thereafter, a laboratory study from the United States showed that vaccination of *Pe. leucopus*, previously infected with *Bo. burgdorferi* s.s. via tick bite, with recombinant OspA resulted in reduced acquisition of spirochetes by feeding xenodiagnostic *Ix. scapularis* larvae from the infected mice (Tsao et al., 2001). A subsequent proof-of-concept field study in the northeastern United States demonstrated that vaccination via needle of wild *Pe. leucopus* against *Bo. burgdorferi* s.s. resulted in the odds of infection with *Bo. burgdorferi* s.l. in questing *Ix. scapularis* nymphs being reduced by 32–40% in four of six treatment sites whereas no or little difference was seen in the other two treatment sites (Tsao et al., 2004). The promising result generated substantial interest in the development of oral bait delivery systems to immunize wild rodent reservoirs against *Bo. burgdorferi* s.s. using OspA-based vaccines (Gomes-Solecki et al., 2006; Scheckelhoff et al., 2006; Bhattacharya et al., 2011; Richer et al., 2011; Telford et al., 2011; Voordouw et al., 2013; Kern et al., 2016). This included laboratory studies on *Mu. musculus* (Gomes-Solecki et al., 2006; Scheckelhoff et al., 2006) and *Pe. leucopus* (Bhattacharya et al., 2011; Richer et al., 2011; Voordouw et al., 2013) showing that oral vaccination against *Bo. burgdorferi* s.s. using OspA-based vaccines could clear spirochetes from feeding infected ticks and prevent infection in the immunized mice challenged with these ticks. Two of the studies on *Pe. leucopus* (Bhattacharya et al., 2011; Voordouw et al., 2013) also indicated that although the immunization with OspA does not clear previous infection from an animal (as OspA is not expressed by the spirochetes in the mouse host), it can reduce the efficiency of acquisition by feeding uninfected ticks of *Bo. burgdorferi* s.s. from infected animals. There also is evidence for maternal transfer to pups of neutralizing antibodies to *Bo. burgdorferi* s.s. OspA after oral vaccination of lactating *Mu. musculus* mice (Phillip et al., 2021).

Laboratory studies focused on two different delivery systems for OspA, via vaccinia virus (Scheckelhoff et al., 2006; Bhattacharya et al., 2011; Kern et al., 2016) or *Escherichia coli* (Gomes-Solecki et al., 2006; Richer et al., 2011; Voordouw et al., 2013). Subsequent field studies were conducted for the latter delivery system, first using live *Es. coli* (Richer et al., 2014) and subsequently inactivated *Es. coli* (Stafford et al., 2020). The initial field study was conducted in New York State woodland settings from 2007 to 2011; in this proof-of-concept study, the oral reservoir-targeted vaccine (RTV) bait was produced daily and offered to rodents within Sherman live traps (Richer et al., 2014). *Peromyscus leucopus* from treatment sites were reported to have elevated levels of anti-OspA antibodies compared to mice from control sites, but no data were presented for detection of active infection with *Bo. burgdorferi* s.l. in the animals. The prevalence of infection with *Bo. burgdorferi* s.l. in questing *Ix. scapularis* nymphs decreased successively over the multi-year study, with a 76% reduction recorded after five consecutive treatment years. However, actual *Bo. burgdorferi* s.l. infection rates of 25 to 45% were still recorded for questing nymphs in the four treatment plots two years after the intervention was started and an overall reduction >25% in infection prevalence was not observed until three years after the RTV bait was first deployed. The slowly building impact on infected questing nymphs across study years was not surprising as the percentage of *Pe. leucopus* in the treatment plots that were considered to have achieved protective antibody levels were low, ranging from 10 to 33%.

Follow-up work conducted by US Biologic, Inc. (Memphis. TN, USA) focused on developing an inactivated field deployable formulation for the oral RTV bait. The palatability of an experimental rodent pellet formulation, coated with Rhodamine B dye to mark the rodents consuming the pellets, was confirmed for *Pe. leucopus* in a residential setting in Connecticut (Williams et al., 2020). High proportions (≥80%) of trapped mice were found to have consumed the pellets regardless of whether they were spread by hand or offered in PROTECTA Sidekick^®^ boxes (Bell Laboratories, Inc.) or a specially designed timed-release bait station (LymeShield bait station, US Biologic, Inc.). Moreover, an inactivated formulation of the oral RTV delivered in the bait pellet was evaluated on residential properties in Connecticut over a two-year period (Stafford et al., 2020). The RTV bait was deployed in PROTECTA Sidekick^®^ boxes distributed along lawn-woods ecotones (with boxes spaced ~9 m apart) on treatment properties; boxes were active from late May to mid-August of 2015 and 2016, and RTV bait was refilled as needed based on regular box inspections. Control properties had no boxes or RTV bait deployed. In the second RTV deployment year, there was a statistically significant but modest (less than two-fold) reduction on treatment properties for the percentage of *Pe. leucopus* infected with *Bo. burgdorferi* s.l., and a stronger, approximately three-fold, reduction for the percentage of *Pe. leucopus* from which ≥1 *Bo. burgdorferi* s.l.-infected feeding *Ix. scapularis* larva was recovered. The study did not present data for prevalence of infection with *Bo. burgdorferi* s.l. in questing *Ix. scapularis* nymphs, which would have been more informative to assess the risk for humans encountering infected ticks. The oral RTV bait from US Biologic, Inc. used by Stafford et al. (2020) is in the process of being registered as a commercial product by the United States Department of Agriculture (J. van Oosterwijk, US Biologic, Inc.; personal communication). Modeling approaches have been used to explore the impact of an RTV on the prevalence of infection with *Bo. burgdorferi* s.s. in questing *Ix. scapularis* nymphs and human Lyme disease cases (Tsao et al., 2012; Voordouw et al., 2013), as well as to conduct a cost-benefit analysis for the intervention (Carrera-Pineyro et al., 2020). These models generated outputs in favor of using the RTV but with one notable caveat underscored by Tsao et al. (2012): the impact of the RTV is strongly influenced by the proportion of larval ticks that feed on *Peromyscus* mice versus other hosts. As the abundance of *Peromyscus* mice can differ greatly between localities as well as between years in the same locality, substantial variation should be expected in the performance of the intervention across space and time. Consequently, when a field deployable oral RTV bait comes on the market, studies will need to be conducted in different ecological settings to assess its efficacy to reduce *Bo. burgdorferi* s.l. infection not only in *Peromyscus* mice and *Ix. scapularis* larvae collected while feeding on these rodents, but also in questing *Ix. scapularis* nymphs posing a threat to bite humans.

### 3.7. Genetically engineered white-footed mice refractory to Bo. burgdorferi s.s

One very different rodent-targeted approach to reduce the intensity of enzootic pathogen transmission is to genetically engineer and release *Pe. leucopus* that are refractory to *Bo. burgdorferi* s.s. (Buchtal et al., 2019). “Mice against ticks” is described as an ecological engineering project aiming to use CRISPR-based genome editing to heritably immunize *Pe. leucopus* against this pathogen. In the early stage of the project, described by Buchtal et al. (2019), the primary focus was on community engagement in prospective intervention areas (islands off the coast of Massachusetts) to gage the acceptance of various technical options, including different methods to heritably immunize the mice and introduce them into the environment. Part of the challenge is finding a middle ground between continuous inundative release of genetically engineered mouse individuals (high cost) and release of mice with a self-propagating CRISPR gene drive (potential for uncontrollable spread).

## 4. What is the future of rodent-targeted approaches to suppress pathogen-infected *Ix. scapularis*?

Despite the challenges described for different rodent-targeted approaches in Section 3, the general concept of suppressing *Ix. scapularis* and its associated human pathogens by targeting rodents serving as important tick hosts and pathogen reservoirs remains a logical and promising means of complementing methods that are based on attacking the tick while it is actively seeking a host, such as using an acaricide spray, or making the environment less favorable for the tick to survive, such as using landscaping and vegetation management techniques to reduce moist microhabitats on residential properties. Due to the sensitivity of rodent-targeted approaches to the presence of alternative tick hosts and pathogen reservoirs not impacted by these interventions, they are better suited to be part of integrated tick management approaches rather than being used as stand-alone interventions, unless the host community in the intervention area is known to be strongly dominated by rodent species expected to be impacted by the implemented rodent-targeted control method. When implemented as stand-alone interventions, both types of approaches to treat rodents topically with acaricides (see Sections 3.1 and 3.2.) have generated highly variable results for impact on the density of host-seeking *Ix. scapularis* nymphs. The reasons underlying the observed variation in impact are not fully understood and, consequently, the specific circumstances under which this rodent-targeted approach will have the desired level of impact remain unclear. Additional studies are needed to optimize and standardize implementation of different rodent-targeted approaches to the point where a robust impact is ensured across a wide range of habitat types and tick host communities. These efforts also need to consider the cost of the implementation, which for example will rise with increasing density of deployment units, or regular replacement or bait replenishment of depleted units, to ensure that all individuals of the targeted rodent species are adequately treated. Current estimates for the willingness of homeowners in Lyme disease endemic areas to pay for rodent-targeted property treatment to control ticks indicate that most (76%) of those saying they are willing to use rodent-targeted tick control are not willing to pay more than $100 per year (Niesobecki et al., 2022), which comes close to covering the cost for deployment of Damminix Tick Tubes^®^/Thermacell^®^ Tick Control Tubes but falls far short of the cost for deployment of SELECT TCS bait boxes (Jordan and Schulze, 2019). Cost estimates are not yet available for emerging approaches, such as the rodent-targeted vaccine against *B. burgdorferi* s.s. (see Section 3.6) and oral acaricides for use in rodents (see Section 3.3). In conclusion, rodent-targeted approaches remain promising components of integrated tick management but there are concerns about the robustness of their impact across habitats and tick host communities as well as the implementation cost in relation to what homeowners say they are willing to pay for tick control.

## Figures and Tables

**Table 1:** Characteristics of different rodent-targeted approaches to reduce acarological risk of human encounters with pathogen-infected *Ixodes scapularis* ticks.

Approach	Projected timeline for commercial products	Expected impacts on rodent pathogen reservoirs			Expected impacts on questing *Ix. scapularis*	
		Reduced abundance	Reduced tick infestation	Reduced pathogen infection prevalence	Reduced abundance	Reduced pathogen infection prevalence
Topically delivered acaricide	Available now^c^	No	Yes	Yes (multiple pathogens)	Yes	Yes (multiple pathogens)
Orally delivered acaricide	1–2 years^d^	No	Yes	Yes (multiple pathogens)	Yes	Yes (multiple pathogens)
Orally delivered antibiotic	Unclear	No	No	Yes (multiple pathogens)	No	Yes (multiple pathogens)
Orally delivered anti-tick vaccine	Unclear	No	Yes	Yes (multiple pathogens)	Yes	Yes (multiple pathogens)
Orally delivered vaccine against *Borrelia burgdorferi* sensu stricto^a^	1 year^e^	No	No	Yes (only *Borrelia burgdorferi* sensu stricto)	No	Yes (only *Borrelia burgdorferi* sensu stricto)
Release of engineered white-footed mice with heritable refractoriness to *Borrelia burgdorferi* sensu stricto^b^	Unclear	Yes^f^	No	Yes (only *Borrelia burgdorferi* sensu stricto)	No	Yes (only *Borrelia burgdorferi* sensu stricto)

a Developed against *Borrelia burgdorferi* sensu stricto and not yet evaluated against other species within the *Borrelia burgdorferi* sensu lato complex.

b Presumably primarily targeting *Borrelia burgdorferi* sensu stricto.

c Damminix Tick Tubes^®^ (Ecohealth, Inc., Brookline, MA, USA), Environmental Protection Agency registration number 56783–1; Thermacell^®^ Tick Control Tubes (Thermacell Repellents, Inc., Bedford, MA, USA), Environmental Protection Agency registration number 71910–10; SELECT TCS Tick Control System or TICK BOX^™^ Tick Control System (Tick Box Technology Corporation, Norwalk, CT, USA), Environmental Protection Agency registration number 85306–1.

d Product in the process of being registered for commercial use by Genesis Laboratories, Inc. (Wellington, CO, USA).

e Product in the process of being registered for commercial use by US Biologic, Inc. (Memphis, TN, USA).

f Decreased proportion of white-footed mouse individuals present within release area capable of serving as reservoirs of *Borrelia burgdorferi* sensu stricto.

**Table 2:** Characteristics of key field studies from the northeastern United States (Connecticut, New Jersey, and New York) on topical acaricide treatment of rodents via bait boxes to suppress *Ixodes scapularis* and reduce the intensity of enzootic transmission of human disease agents. All studies included bait box deployment in both spring (targeting nymphal ticks) and summer (targeting larval ticks) during at least one treatment year.

	Study environment^b^		Design characteristics of field intervention				Outcome measures presented for rodents, *Ix. scapularis* and humans								
							Rodents				Questing ticks		Humans		
Bait box	Treatment	Control^c^	Bait box placement on treated residential properties	Rebaiting of deployed boxes	Treatment years	Pretreatment data for outcome measures	Abundance	Bait box use^g^	Tick infestation	Pathogen infection^h^	Abundance	Pathogen infection^h^	Tick encounters	Tick-borne disease^h^	Reference
Prototype	RP (*n* = 154)	W (*n* = 5)	Boxes spaced 10 m apart in row along ecotone of maintained landscaping (mostly lawn) with woods, brush or stone walls; extra boxes placed near wood-piles and outbuildings.	Yes	1999–2001^d^	Yes^e^	Yes^f^	Yes	Yes	Yes	Yes	Yes	No	No	Dolan et al. (2004)
Maxforce TMS	RP (*n* = 4)	RP (*n* = 3); W (*n* = 1)	Boxes spaced 25 m apart in rows (i) along lawn ecotone with woods and (ii) 10 m into wooded habitat	Yes	2008–2009	Yes^e^	No	Yes	Yes	N/A^i^	Yes	n/a ^i^	No	No	Dolan et al. (2017)
SELECT TCS^a^	RP (*n* = 12)	W (*n* = 1)	Boxes spaced 10 m apart in rows located (i) 3 m into woods from ecotone with lawn and (ii) 13 m into wooded habitat (as needed depending on extent of wooded habitat on the property)	No	2012–2013	Yes^e^	Yes^f^	Yes	Yes	No	Yes	No	No	No	Schulze et al. (2017)
SELECT TCS^a^	RP (*n* = 6)	W (*n* = 1)	Boxes spaced 20 m apart in rows located (i) 10 m into woods from ecotone with lawn and (ii) 30 m into wooded habitat (as needed depending on extent of wooded habitat on the property)	No	2014–2015	Yes^e^	Yes^f^	Yes	Yes	No	Yes	No	No	No	Jordan and Schulze (2019)
SELECT TCS^a^	RP (*n* = 269)	RP (*n* = 269)	Boxes spaced 10 m apart in row located 3 m into woods or brush from ecotone with lawn, with additional boxes placed along unsealed rock piles and wood piles located at or near the wooded edge	No	2012–2014; or 2013–2015	Yes^e^	No	No	No	No	Yes	Yes	Yes	Yes	Hinckley et al. (2021)
SELECT TCS^a^	RP (n~220 RPs in six neighborhood clusters)	RP (n~220 RPs in six neighborhood clusters)	Boxes placed ≥10 m apart in all habitat types sampled for ticks; deployed in protected locations (e.g., along building foundations and under vegetation)	No	2017–2020	No	No	No	Yes	No^j^	Yes	No^j^	Yes	Yes	Keesing et al. (2022)

a SELECT TCS product label recommendation language for box deployment: “Boxes are placed a minimum of 10 m (30 feet) apart near the edge of maintained landscaping and woodlots and/or brush. For woodlots that extend farther than 40 feet an additional row of units should be considered for maximum control. The second row should be placed 30–40 feet from the first set of boxes.”.

b RP, residential properties; W, woodland sites.

c Control areas without bait boxes (Dolan et al., 2004, 2017; Schulze et al., 2017; Jordan and Schulze, 2019) or with placebo bait boxes lacking fipronil (Hinckley et al., 2021; Keesing et al., 2022).

d A single treatment year for the majority of properties (*n* = 110), with smaller numbers treated over 2 years (*n* = 31) or three years (*n* = 13).

e Including collection of questing nymphs in the year the treatment started as nymphs of that cohort (resulting from larvae fed on hosts in the summer of the previous year) were not considered to be impacted by the treatment in the first year.

f Based on data presented for both number of rodents captured and number of trap days/nights.

g Based on removal of bait from the boxes.

h Pathogens and diseases associated with *Ix. scapularis*, including *Borrelia burgdorferi* sensu lato (Lyme disease), *Anaplasma phagocytophilum* (anaplasmosis), and *Babesia microti* (babesiosis).

i Not applicable; only measures related to tick infestation of rodents or abundance of questing ticks is considered here as the intervention also included antibiotic treatment of the rodents which would impact pathogen infection in both rodents and questing ticks.

j Data were collected but not included in this publication.

## Data Availability

No data was used for the research described in the article.
